# The Use of Bilberry Leaves (*Vaccinium myrtillus* L.) as an Efficient Adsorbent for Cationic Dye Removal from Aqueous Solutions

**DOI:** 10.3390/polym14050978

**Published:** 2022-02-28

**Authors:** Giannin Mosoarca, Cosmin Vancea, Simona Popa, Mircea Dan, Sorina Boran

**Affiliations:** Faculty of Industrial Chemistry and Environmental Engineering, Politehnica University Timisoara, Bd. V. Parvan, No. 6, 300223 Timisoara, Romania; mircea.dan@upt.ro (M.D.); sorina.boran@upt.ro (S.B.)

**Keywords:** adsorption, lignocellulosic material, equilibrium, kinetics, thermodynamic, Taguchi optimization

## Abstract

In this study, a new lignocellulosic bioadsorbent, bilberry (*Vaccinium myrtillus* L.) leaves powder, was used to remove the methylene blue dye from aqueous solutions. The characterization of the adsorbent was performed by FTIR, SEM and color analysis. The influence of pH, contact time, adsorbent dose, initial dye concentration, temperature and ionic strength on the adsorption process were followed. Equilibrium, kinetic, and thermodynamic studies were conducted in order to understand the adsorption process mechanism. Process optimization was performed using the Taguchi method. Sips isotherm and general order kinetic model characterize the adsorption process. The maximum adsorption capacity, 200.4 (mg g^−1^), was better compared with other similar bioadsorbents. Thermodynamic parameters indicated that the adsorption process is spontaneous, favorable and endothermic and also that physisorption is involved in the process. The factor with the highest influence on the dye removal process was pH, followed by contact time, temperature, adsorbent dose, ionic strength and initial dye concentration. The obtained results revealed that the bioadsorbent material based on bilberry (*Vaccinium myrtillus* L.) leaves is highly efficient for cationic dyes removal from aqueous solutions.

## 1. Introduction

Nowadays, dyes have many applications in everyday life and are widely used in various industries such as textile, pigments, plastics, printing, leather, food, rubber, paper and cosmetics [1,2,3,4,5,6,7]. Most of these dyes end up in wastewater which, without proper treatment, can cause serious environmental problems [5,7,8,9,10,11].

Cationic dyes are an important category of dyes used in industry, some of which having high toxicity. More than 10% of cationic dyes produced annually reach in wastewaters [12,13]. Methylene blue is a cationic dye commonly used in the textile industry, especially for dyeing cotton, silk and wool [1,2,14,15]. It is used in medical practice for treatment of methaemoglobinemia and cyanide poisoning and can also play the role of staining agent in diagnostic examinations [2,16]. It may have a harmful effect on human health leading to eye irritation, vomiting, diarrhea, tachycardia and respiratory problem [15,16,17].

Adsorption is a commonly used method of removing dyes from wastewater because it has high efficiency, selectivity and flexibility, design simplicity, ease of operation and low economic costs. Another major advantage of this process is the wide variety of adsorbent materials available. The identification and use of new adsorbent biomaterials that are easily available in nature in large quantities leads to a pronounced decrease in costs and represent a topical issue [16,18,19,20,21,22].

Due to their chemical composition, natural plant-based materials and agricultural waste have been successfully tested for the adsorption of various organic and inorganic pollutants [23,24,25]. Plant leaves contain cellulose, lignin, polyphenolics, plant pigments and protein that can provide active adsorption sites for dye retention [26,27,28]. In addition, these materials are available in large quantities and do not require additional preliminary stages of treatment and activation. Thus, the costs are reduced and most of the time the regeneration of these adsorbents is not necessary [29,30].

Bilberry (*Vaccinium myrtillus* L.) is a low-growing shrub native to Europe, wide-spread in mountainous and sub-montane areas [31]. It is found all over the world, especially in temperate and sub-arctic regions [32]. Dark purple fruits can be eaten fresh or processed and contain many vitamins and minerals. They also contain an impressive amount of antioxidants and phenolic compounds, necessary for maintaining health, and which give them antioxidant, antiviral and antibacterial properties [33,34,35,36]. Not only the fruits are beneficial to human health but also various parts of the plant, which have been used in traditional medicine in Europe for over 1000 years [32,33,37].

The aim of this study was to remove methylene blue cationic dye from aqueous solutions using the new bioadsorbent material obtained from bilberry (*Vaccinium myrtillus* L.) leaves. After characterizing the bioadsorbent, using FTIR, SEM and color analysis, the influence of pH, adsorbent dose, ionic strength, contact time, initial dye concentration and temperature on the adsorption process were followed. Equilibrium, kinetics, and thermodynamics studies were also performed.

## 2. Materials and Methods

The bilberry (*Vaccinium myrtillus* L.) dried leaves were purchased from a manufacturer who processes medicinal plants, StefMar (Ramnicu Valcea, Romania). The leaves were washed with distilled water, dried in an air oven at 105 °C for 24 h and then ground electrically. The obtained powder material was passed through a 2 mm sieve. 

In order to characterize the bioadsorbent material, before and after dye adsorption, SEM analysis and FTIR spectroscopy were carried out using a Quanta FEG 250 (FEI, Eindhoven, The Netherlands) scanning electron microscope (1600× magnitude) and a Shimadzu Prestige-21 FTIR (Shimadzu, Kyoto, Japan) spectrophotometer. The colorimetric analysis was recorded for the D65 (natural light) under 10° observer angle, using a Cary-Varian 300 Bio UV-VIS colorimeter (Varian Inc., Mulgrave, Australia) with integrating sphere and a Spectralon standard. All CIEL*a*b* color data were expressed by L*, a*, b* coordinates, where L* corresponds to lightness; a* corresponds to the transition from green (−a*) to red (+a*); and b* corresponds to the transition from blue (−b*) to yellow (+b*). The pH at which the net charge of the adsorbent surface is zero, (PZC), was determined using the solid addition method [38]. 

The adsorption experiments were performed at a constant stirring intensity provided by a shaker. Three independent replicates were made for each experiment. The pH adjustment 0.1 N NaOH and HCl solutions were used. The influence of ionic strength was follow using NaCl as background electrolyte. The methylene blue concentration was measured with a Specord 200 PLUS UV-VIS (Analytik Jena, Jena, Germany) spectrophotometer at a wavelength of 664 nm. 

The dye amounts adsorbed at equilibrium (*q_e_*) and the methylene blue removal percentage *R*(%) were calculated with Equations (1) and (2) [18,38]:

(1) $$q_{e}=\frac{(C_{0}-C_{e})\times V}{m},$$

(2) $$R(\%)=\frac{\left(C_{0}-C_{e}\right)}{C_{0}}\times 100,$$

where: *C*_0_ is the initial dye concentration; *C_e_* is the dye equilibrium concentration; *V* is the solution volume and *m* is the mass of bioadsorbent.

Non-linear equations of pseudo-first order, pseudo-second order, Elovich, general order and Avrami kinetic models were used to analyze the adsorption kinetics. The equations are described below:

(3) $$\mathrm{Pseudo}-\mathrm{first}-\mathrm{order}\;\mathrm{model}\;\mathrm{equation}:\;q_{t}=q_{e}(1-exp^{-k_{1}\times t}),$$

(4) $$\mathrm{Pseudo}-\sec\mathrm{ond}-\mathrm{order}\;\mathrm{model}\;\mathrm{equation}:\;q_{t}=\frac{k_{2}\times t\times q_{e}^{2}}{1+k_{2}\times t\times q_{e}},$$

(5) $$\mathrm{Elovich}\;\mathrm{model}\;\mathrm{equation}:\;q_{t}=\frac{1}{a}ln(1+a\times b\times t),$$

(6) $$\mathrm{General}\;\mathrm{order}\;\mathrm{model}\;\mathrm{equation}:\;q_{t}=q_{n}-\frac{q_{n}}{{\left[k_{n}\times {\left(q_{n}\right)}^{n-1}\times t\times (n-1)+1\right]}^{1/1-n}},$$

(7) $$\mathrm{Avrami}\;\mathrm{model}\;\mathrm{equation}:\;q_{t}=q_{AV}[1-exp{\left(-k_{AV}\times t\right)}^{n_{AV}}],$$

where: *q_t_* is the crystal violet amount adsorbed at time *t*; *k_1_*, *k_2_*, *k_n_* and *k_AV_* are the rate constants of pseudo-first-order, pseudo-second-order, general order and Avrami kinetic models; *q_e_*, *q_n_* and *q_AV_* are the theoretical values for the adsorption capacity; *a* is the desorption constant of Elovich model; *b* is the initial velocity; *n* is the general order exponent and *n_AV_* is a fractional exponent [18,39,40,41,42,43].

Non-linear equations of Langmuir, Freundlich, Temkin, Sips and Redlich-Peterson isotherms were used to describe the adsorption equilibrium. The corresponding equations are as follows:

(8) $$\mathrm{Langmuir}\;\mathrm{isotherm}\;\mathrm{equation}:\;q_{e}=\frac{q_{m}\times K_{L}\times C_{e}}{1+K_{L}\times C_{e}},$$

(9) $$\mathrm{Freundlich}\;\mathrm{isotherm}\;\mathrm{equation}:\;q_{e}=K_{F}\times C_{e}^{1/n_{F}},$$

(10) $$\mathrm{Temkin}\;\mathrm{isotherm}\;\mathrm{equation}:\;q_{e}=\frac{R\times T}{b}\times ln(K_{T}\times C_{e}),$$

(11) $$\mathrm{Sips}\;\mathrm{isotherm}\;\mathrm{equation}:\;q_{e}=\frac{Q_{sat}\times K_{S}\times C_{e}^{n}}{1+K_{S}\times C_{e}^{n}},$$

(12) $$\mathrm{Redlich}-\mathrm{Peterson}\;\mathrm{isotherm}\;\mathrm{equation}:\;q_{e}=\frac{K_{RP}\times C_{e}}{1+a_{RP}\times C_{e}^{\beta_{RP}}},$$

where: *q_m_* and *Q_sat_* are the maximum absorption capacities; *K_L_*, *K_F_*, *K_T_*, *K_S_* and *K_RP_* are the Langmuir, Freundlich, Temkin, Sips and Redlich-Peterson isotherms constants; *1/n_F_* is an empirical constant indicating the intensity of adsorption; *R* is the universal gas constant; *T* is the absolute temperature; *b* is Temkin constant which related to the adsorption heat; *n* is Sips isotherm exponent; *a_RP_* is Redlich-Peterson isotherm constant and *β_RP_* is Redlich-Peterson exponent which can vary between 0 and 1 [18,39,40,41,42,43]. 

In order to establish the proper isotherm and kinetic models values of determination coefficient (*R*^2^), sum of square error (SSE), chi-square (*χ*^2^) and average relative error (ARE) were calculated [43]. The criterion for their applicability was the higher value for *R*^2^ and the lower values for *SSE*, *χ*^2^, and *ARE* was the criterion for establishing the best model.

(13) $$R^{2}=1-\frac{\sum_{i=1}^{n}{\left(y_{i,exp}-y_{i,mod}\right)}^{2}}{\sum_{i=1}^{n}{\left(y_{i,exp}-\bar{y_{i,exp}}\right)}^{2}},$$

(14) $$SSE=\sum_{i=1}^{n}{\left(y_{i,exp}-y_{i,mod}\right)}^{2},$$

(15) $$\chi^{2}=\sum_{i=1}^{n}\frac{{\left(y_{i,exp}-y_{i,mod}\right)}^{2}}{y_{i,mod}},$$

(16) $$ARE=\frac{100}{n}\sum_{i=1}^{n}\left|\frac{y_{i,exp}-y_{i,mod}}{y_{i,mod}}\right|,$$

where: *y_i,exp_* is the experimental value; *y_i,mod_* is the modeled value; $\bar{y_{i,exp}}$ is the mean values and *n* is the total amount of information.

The values for Gibbs free energy change, enthalpy change and entropy change were calculated (at 278, 287, 297, 303 and 311 K) using the Equations (17) and (18) [18,40]:

(17) $$\Delta G^{0}=-RTlnK_{L},$$

(18) $$lnK_{L}=\frac{\Delta S^{0}}{R}-\frac{\Delta H^{0}}{RT},$$

where: *R* is the universal gas constant; *K_L_* is the Langmuir constant and *T* is the absolute temperature.

The optimum dye removal conditions were determined using the Taguchi methods, applying the L25 orthogonal array with six factor at five levels (Table 1). The effect of the controllable factors on methylene blue removal efficiency was evaluated by the signal/noise ratio (S/N) analysis choosing the “larger is the better” option [44,45]. The results of the Taguchi method were evaluated by analysis of variance analysis (ANOVA) with which established the percentage contribution of each factor on the dye removal efficiency. All calculations were performed with the Minitab 19 Software (version 19.1.1, Minitab LLC, State College, PA, USA).

Three regenerating agents were used in the desorption studies (0.1 N HCl, distilled water and 0.1 N NaOH). The experiments were performed at a constant stirring rate, for 2 h. The methylene blue desorption percentage *D*(%) was determined with the equation:

(19) $$D(\%)=\frac{m_{d}}{m_{a}}\times 100,$$

where: *m_d_* is the dye amount liberated by the regenerating agent and *m_a_* is the dye amount adsorbed on the adsorbent material.

## 3. Results and Discussion

### 3.1. Bioadsorbent Characterization

**Figure 1:**
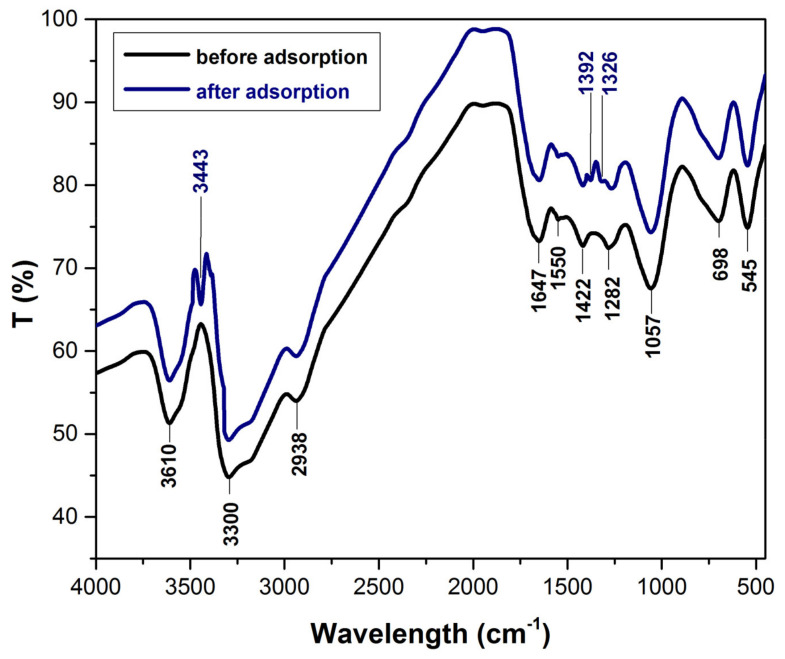
The FTIR spectra (before and after adsorption process) of bioadsorbent obtained from bilberry (*Vaccinium myrtillus* L.) leaves.

The FTIR spectra (Figure 1), before and after adsorption process, reveal that cellulose, hemicellulose and lignin are the main components of the bioadsorbent material. The specific peaks that indicate the functional groups presence of this components are as follow: 3610 cm^−1^ can be attributed to the -OH stretch, free hydroxyl [46], 3300 cm^−1^ can be assigned the –OH stretching vibrations of cellulose, lignin or hemicellulose present in lignocellulosic biomasses [47], 2938 cm^−1^ correspond to CH_2_ stretching vibration [48,49], 1647 cm^−1^ indicate –C=O stretching characteristic of lignin or hemicellulose [50,51], 1550 cm^−1^ can be attributed to the amide II groups [52,53], 1422 cm^−1^ corresponding to –C–H deformation in lignin [54,55], 1282 cm^−1^ can be attributed to the CH deformation in cellulose I and cellulose II [56], 1057 cm^−1^ can be assigned to C–O–C stretching of cellulose [27,39], 698 cm^−1^ were mainly due aromatic out of plane C–H bending vibrations [57,58], 542 cm^−1^ can be attributed C–H bend [59]. After adsorption, FTIR spectra shows some changes and three methylene blue characteristic peak appear that indicate the presence of dye at adsorbent surface: 3443 cm^−1^ corresponds to –NH/–OH overlapped stretching vibration [60], 1392 cm^−1^ can be attributed to the vibration of C–N in the –N(CH_3_)^2+^ group and 1326 cm^−1^ can be associated with the –CH_3_ group [15,61].

**Figure 2:**
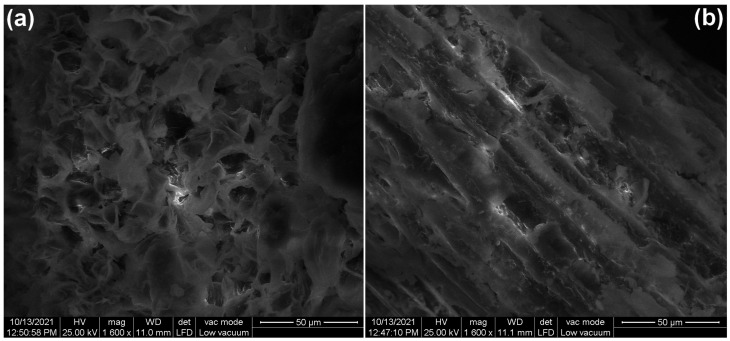
The SEM images of bioadsorbent obtained from bilberry (*Vaccinium myrtillus* L.) leaves: (**a**) before adsorption, (**b**) after adsorption.

The SEM images of adsorbent material are illustrated in Figure 2. Prior to adsorption (Figure 2a), the surface of the adsorbent is heterogeneous, with many pores, folds and voids of various shapes suggesting a suitable structure for the adsorption of dyes. After adsorption (Figure 2b), the surface becomes more homogeneous and compact, which indicate that dye molecules filled the pores and cavities.

**Figure 3:**
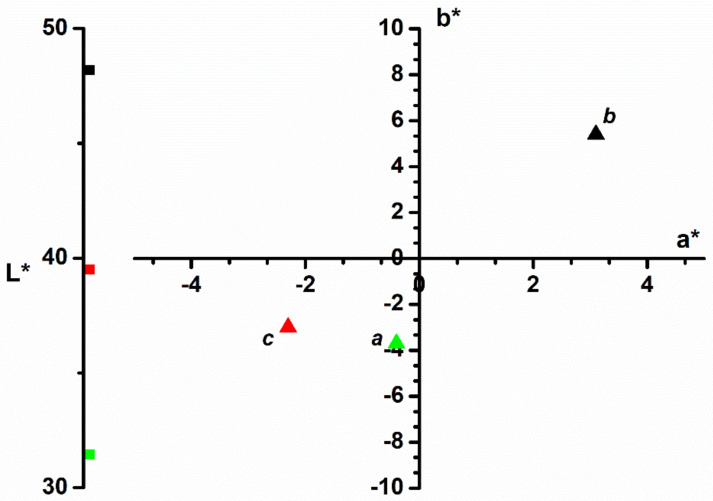
CIEL*a*b* color parameters of: *a*—methylene blue dye; *b*—bilberry leaves before adsorption process; *c*—bilberry leaves after adsorption process.

The color of adsorbent materials (the bilberry leaves powder) that were used in the adsorption process of methylene blue was monitored with the CIEL*a*b* color parameters (Figure 3). The color of vegetables is primarily given by the pigments in the plant. By drying, the green color of live leaves may diminish, but there will still be a residual color of the vegetable wastes (Figure 3, point *b*). 

The color’s behavior during the adsorption process reveals that the dye color passes from the solution onto the adsorption material. Although the bilberry leaves’ luminosity lowers with approximately 10 units after adsorption, the other CIEL*a*b* color parameters namely a* and b* obviously change. Thus, the a* parameter moves from red to green and the b* parameter from yellow to blue. As a result, the color of the bilberry leaves used as adsorbent will turn from light orange (Figure 3, point *b*) to turquoise (Figure 3, point *c*), color similar to the methylene blue dye (Figure 3, point *a*). The color parameters show that the adsorbent material changes its color, which confirms the dye adsorption on the material surface.

**Figure 4:**
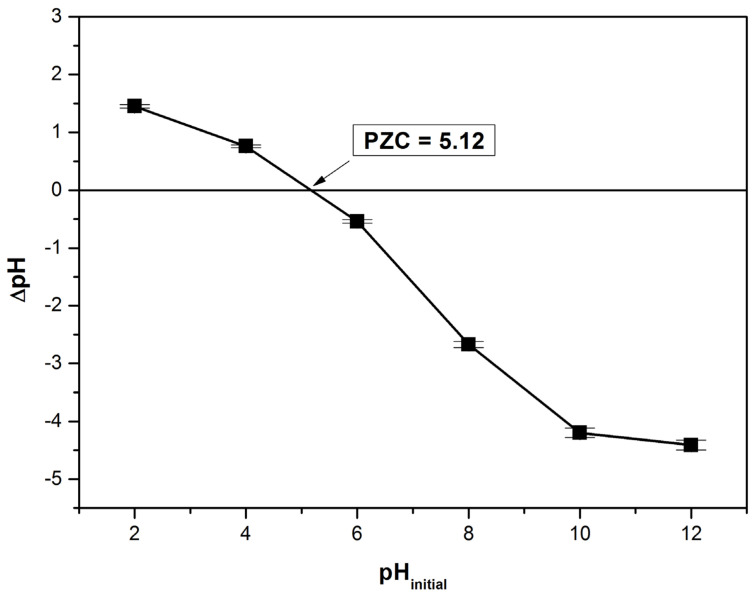
Determination of point of zero charge, PZC, for bioadsorbent material (the solid addition method).

The point of zero charge (PZC) is a significant and useful parameter that indicates when the adsorbent surface has become positively or negatively charged depending on the pH. According to Figure 4 the determined value for this parameter was 5.12. At pH value lower than PZC the adsorbent surface is positively charged and at pH value higher than PZC the adsorbent surface is negatively charged [16,20,27,38]. 

### 3.2. Influence of pH on Dye Adsorption

**Figure 5:**
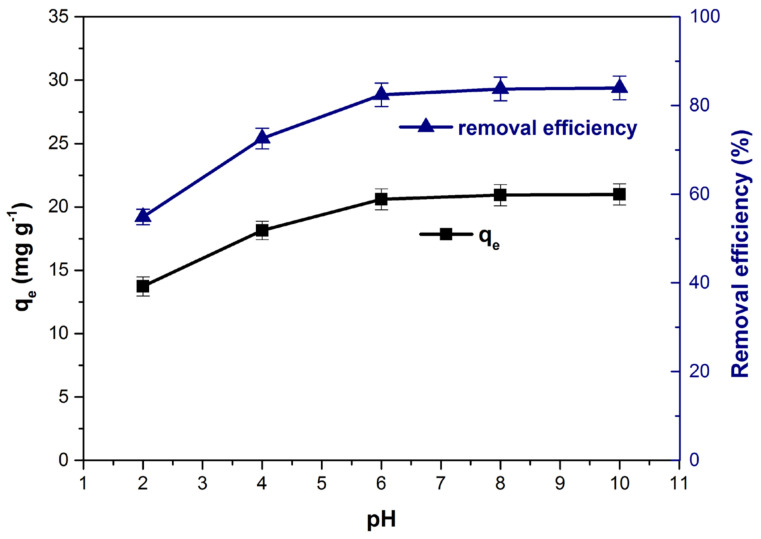
Influence of pH on adsorption capacity and dye removal efficiency.

The Figure 5 shows the influence of the initial solution pH on the adsorption capacity and the removal efficiency. Both parameters recorded the lowest values at pH = 2. As the initial solution pH increased, an increase in adsorption capacity and removal efficiency was observed. Thus, on the pH range 2–6, the adsorption capacity increased from 13.7 (mg g^−1^) to 20.6 (mg g^−1^) and the removal efficiency increased from 54.9% to 82.4%. During the pH range 6–10, the values of the two parameters remained practically constant. Previous studies have reported the same effects of the initial solution pH on the adsorption process [16,62,63]. The low adsorption rate recorded at the lowest values of pH (under PZC) can be explained considering that at these pH values, the adsorbent materials’ surface is positively charged generating an electrostatic repulsion of the cationic dye [1,16]. At higher pH than PZC, the adsorbent surface became negatively charged and favorable conditions for the adsorption of dye cations occur due to the electrostatic attraction [16,20,27,38]. 

### 3.3. Influence of Bioadsorbent Dose on Dye Adsorption

**Figure 6:**
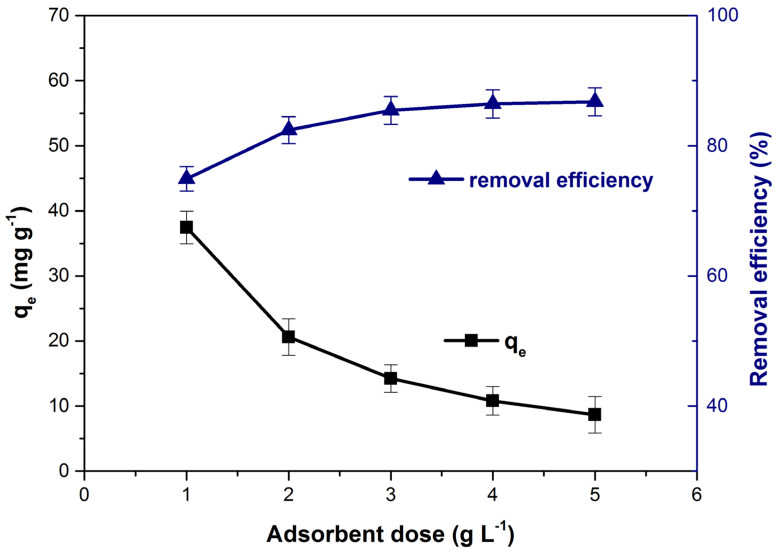
Influence of bioadsorbent dose on adsorption capacity and dye removal efficiency.

The bioadsorbent dose effect on the adsorption capacity and the dye removal efficiency is depicted in Figure 6. The increase of adsorbent dose from 1 to 5 (mg L^−1^), generated an ever-increasing available adsorption sites number which led to an increase in removal efficiency [1,64,65]. Meanwhile, many of this adsorption sites remain unsaturated and aggregation or agglomeration of adsorbent particle may occur [23,66] and the adsorption capacity decrease from 37.46 to 8.67 (mg g^−1^). A similar influence of adsorbent dose on the adsorption capacity and removal efficiency of methylene blue has been previously mentioned by other researchers in their scientific articles [64,65].

### 3.4. Influence of Ionic Strength on Dye Adsorption

**Figure 7:**
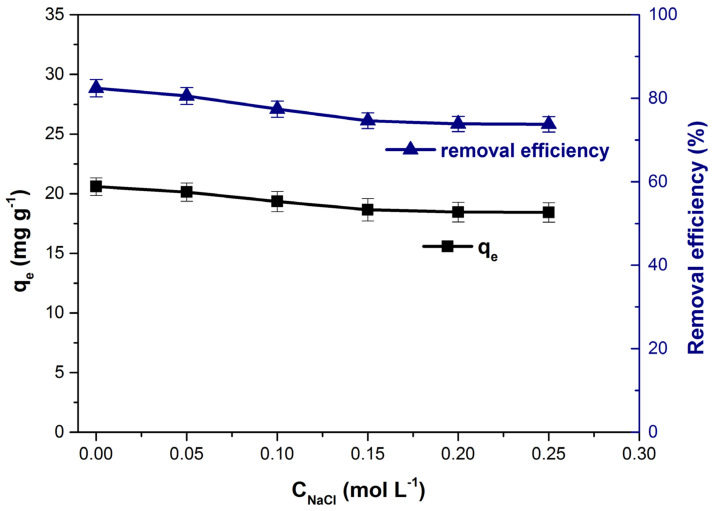
Influence of the ionic strength on adsorption capacity and dye removal efficiency.

The efficiency of the adsorption process can be influenced by the presence of other ions in the solution. In general, colored residual effluents have a high ionic strength due to them. The increase in ionic strength, simulated by the addition of NaCl, led to a decrease in the adsorption capacity and the dye removal efficiency from the solution (Figure 7). The unfavorable effect of ionic strength is due to the competition between dye cations and Na^+^ ions to occupy the free spaces available for adsorption on the adsorbent material surface and has been observed in other adsorption studies regarding the removal of methylene blue from aqueous solutions [38,67,68].

### 3.5. Influence of Contact Time on Dye Adsorption. Process Kinetics

**Figure 8:**
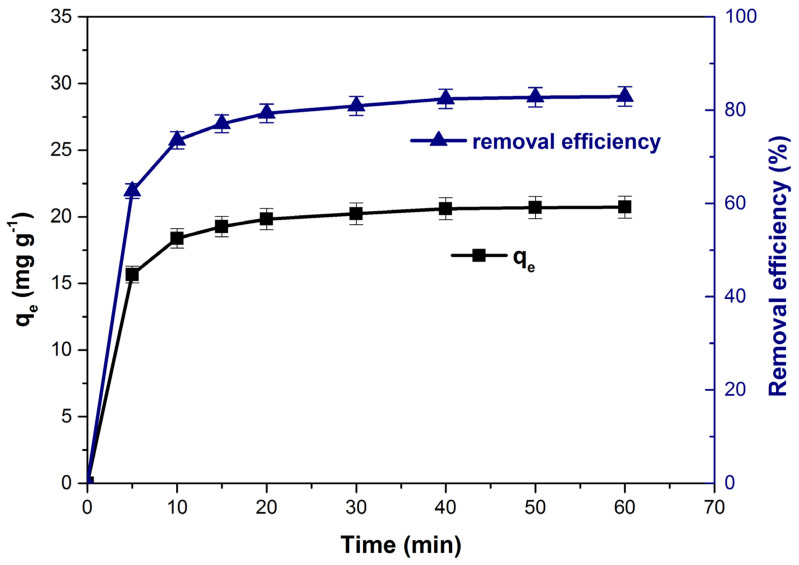
Influence of contact time on adsorption capacity and dye removal efficiency.

The variation of adsorption capacity and dye removal efficiency as function of con-tact time is presented in Figure 8. The values of the monitored parameters increased significantly until reaching equilibrium at a contact time of 40 min.

Initially, the adsorption of the dye was quite fast due to the large number of adsorption sites available on the adsorbent material surface [1,23,69]. Over time, they are gradually occupied by dye molecules and the adsorption capacity and methylene blue removal efficiency increase slowly. After the equilibrium was established, almost the entire adsorbent surface of was covered by the dye molecules [1,24,70] and the variation of the both parameters was insignificant. The equilibrium times obtained for methylene blue adsorption on different similar bioadsorbent materials are summarized in Table 2.

**Figure 9:**
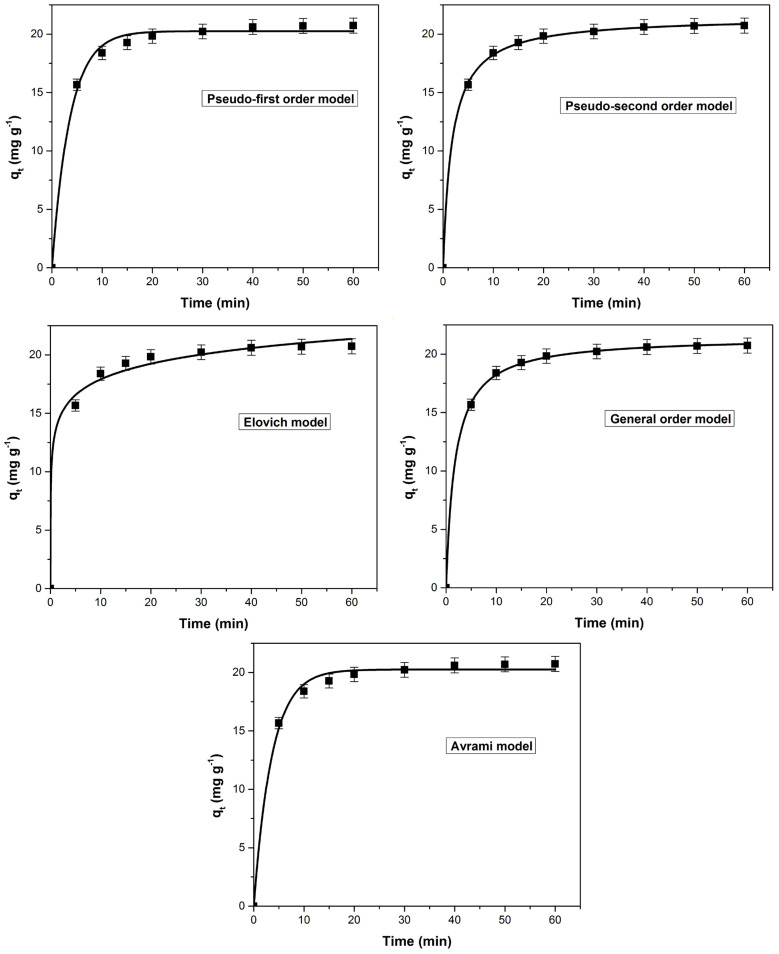
The tested kinetic models (non-linear forms) for the methylene blue adsorption on bioadsorbent obtained from bilberry (*Vaccinium myrtillus* L.) leaves.

The dye adsorption kinetic was evaluated using the following kinetic models (non-linear form): pseudo-first order, pseudo-second order, Elovich, general order and Avrami. Analyzing the curves of kinetic models (Figure 9), constants and the corresponding error functions (Table 3) it can be concluded that the most suitable model is general order. This model assumed that the order of an adsorption process must logically follow the same pattern as in a chemical reaction, where the order is experimentally determined instead of being predicted by a given model [75,76,77,78]. The coefficient of determination (*R*^2^) values for the general order and the pseudo-second models were very close but the lower values obtained for the error functions (SSE, *χ*^2^ and ARE) were the basis for the established conclusion.

### 3.6. Influence of Initial Dye Concentration on Dye Adsorption. Adsorption Isotherms

**Figure 10:**
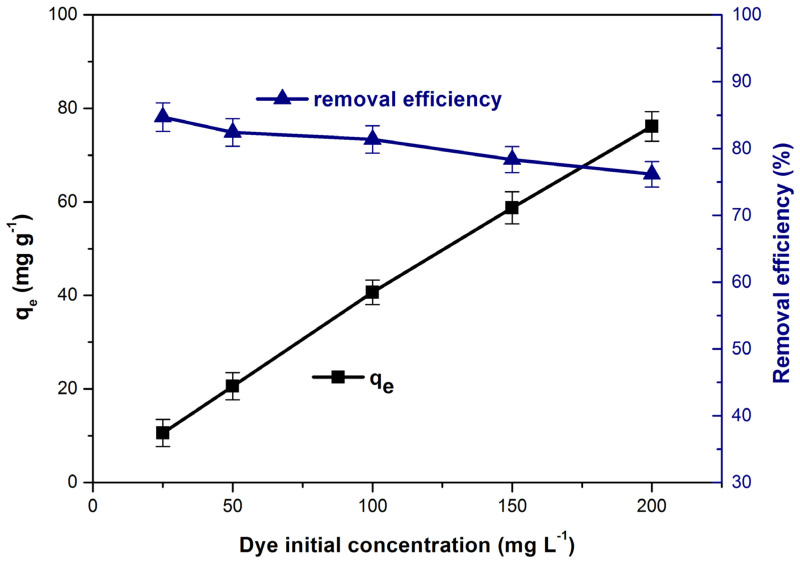
Influence of initial dye concentration on adsorption capacity and dye removal efficiency.

The increase of the initial concentration, from 25 to 200 (mg L^−1^) leads to the appearance of two phenomena that cause the increase of the adsorption capacity from 10.5 to 7.1 (mg g^−1^) (Figure 10). First of all, the concentration gradient between the dye solution and the adsorbent material surface increases, therefore the driving force required to overcome the resistance of the mass transfer through the solid/solution interface increases [1,23,28]. Secondly, the adsorption process is favored by the increase in the collisions number between the methylene blue molecules and the adsorbent material particles [1,25]. At the same time, high concentrations of dye cause the accumulation of molecules on the adsorbent surface and the adsorption sites become saturated which determine a decrease in methylene blue removal efficiency [23,78,79]. Similar results have been reported in other studies on the removal of methylene blue from aqueous solutions by adsorption [23,38].

**Figure 11:**
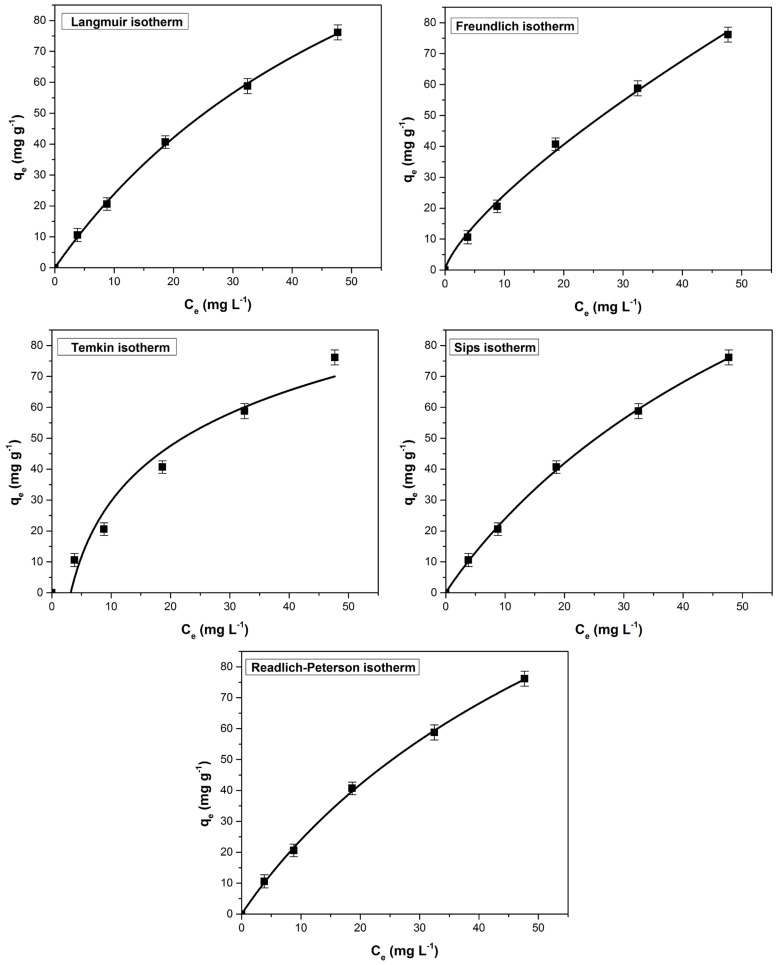
The tested adsorption isotherms (non-linear forms) for the methylene blue adsorption on bioadsorbent obtained from bilberry (*Vaccinium myrtillus* L.) leaves.

The equilibrium adsorption process was assessed using non-linear isotherms Langmuir, Freundlich, Temkin, Sips and Redlich-Peterson. Following the analysis of the fitted isotherm curves (Figure 11), constants and the corresponding error functions (Table 4) it was found that Sips isotherm best describe the process. This model assumes that the process follows the Freundlich model (diffused adsorption) at low methylene blue concentrations and the Langmuir model (monomolecular adsorption) at high concentrations. Scientific literature reported that Sips isotherm best described other processes of methylene blue adsorption from water [80,81,82].

Table 5 compares the maximum adsorption capacities obtained for similar bio-materials used for the methylene blue adsorption. The comparison reveals that our material have a better adsorption capacity than many other studied bioadsorbents.

### 3.7. Influence of Temperature on Dye Adsorption. Process Thermodynamics

**Figure 12:**
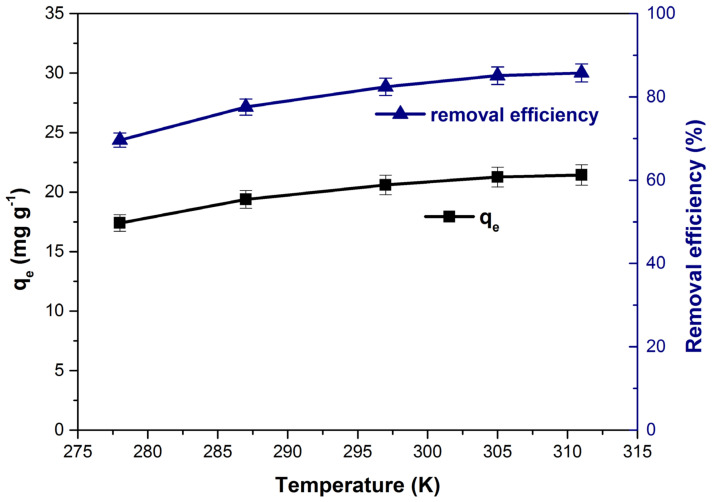
Influence of temperature on the adsorption capacity and dye removal efficiency.

Figure 12 shows the influence of increasing temperature on the adsorption capacity and dye removal efficiency. Both parameters increase with the increase in temperature from 278 to 311 K denoting that the process is endothermic in nature [1,84]. The results are in agreement with results obtained in other previous studies that use bioadsorbents for methylene blue adsorption [27,28,62] and can be explained by reducing the solution’s viscosity as the temperature increases. Thus, increasing dye molecules’ mobility leads to an increase in the diffusion rate in the pores of the adsorbent material [47,84].

**Figure 13:**
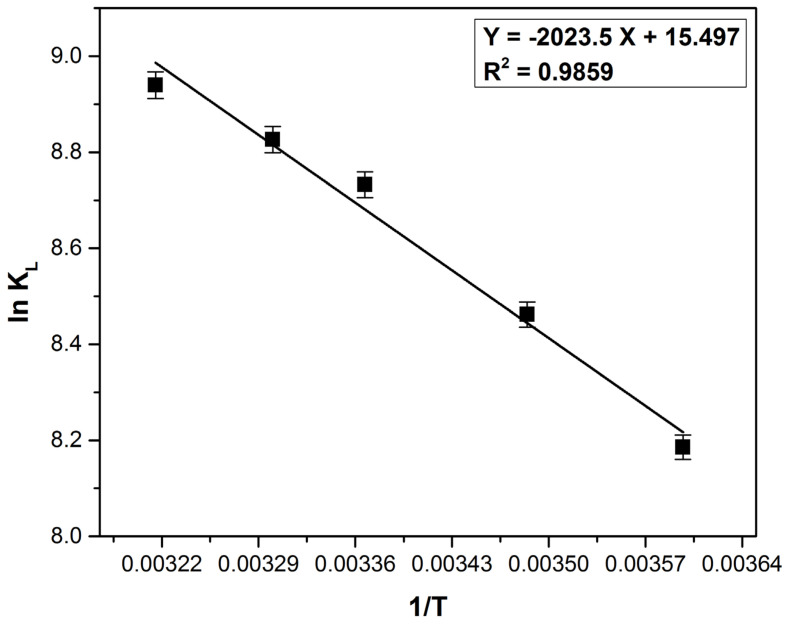
Plot of ln K_L_ vs. 1/T for the methylene blue adsorption on bioadsorbent obtained from bilberry (*Vaccinium myrtillus* L.) leaves.

Analyzing the thermodynamic parameters (Table 6) calculated based on the data obtained from Figure 13, it can be stated that the adsorption process is spontaneous, favorable and endothermic (ΔG^0^ is negative and ΔH^0^ is positive). The positive value of ΔS^0^ indicate an increased degree of disorder at the adsorbent–liquid interface and shows the adsorbent’s affinity for methylene blue. Similar observations were mentioned in other studies which aimed the methylene blue removal on similar bioadsorbents [16,22,62,88]. 

The value of ΔH^0^ lower than 20 (kJ mol^−1^) reveals the physical adsorption presence [89] with van der Waals interaction implied in the process mechanism [84,90]. The physisorption is involved in the process when ΔG^0^ ranges from −20 to 0 (kJ mol^−1^) and the physisorption together with chemisorption is involved when ΔG^0^ ranges from −20 to −80 (kJ mol^−1^) [29,91,92]. The ΔG^0^ value (Table 5) indicates the involvement of physisorption, but a small chemical effect may enhance the process.

### 3.8. Taguchi Optimization

The L25 orthogonal array used in the experiments and results obtained after each run are presented in Table 7. The S/N ratio for the controllable factors along with delta values (the difference between the highest and lowest average response values for each factor) and factors’ significance ranks are summarized in Table 8. The optimum dye removal conditions removal are marked with an asterisk. The factor with the highest influence on the dye removal process is pH, followed by contact time, temperature, adsorbent dose, ionic strength and initial dye concentration. The same order of controllable factor influence was confirmed by ANOVA analysis with which the percentages contribution were also determined (Table 8).

### 3.9. Desorption Study

**Figure 14:**
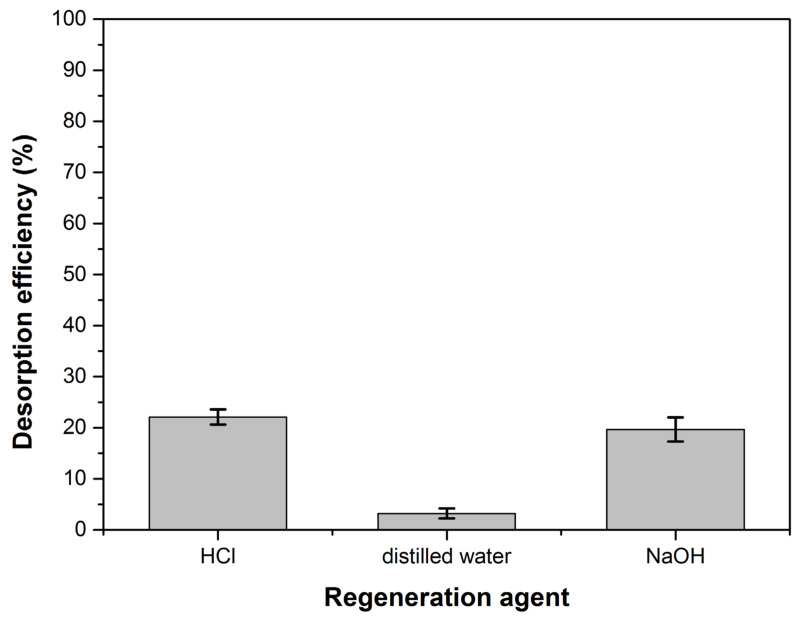
The desorption efficiency for the regeneration agents.

Figure 14 shows the desorption efficiency values for the three regenerating agents used. The very low desorption percent in distilled water shows that the adsorption is not dominated by weak bonds [93]. The desorption results obtained using HCl and NaOH indicate that ion exchange may be involved in adsorption but it doesn’t have a very significant role and other strong forces intervene in process [94,95,96]. These observations are consistent with thermodynamic studies that have shown the involvement of physical adsorption enhanced by a chemical effect in the dye retention process.

HCl and NaOH had approximately the same desorption percentage but its value (below 25%) makes the desorption process not cost-effective, both technically and economically. However, the adsorbent material is easily found in large quantities in nature, so the fact that it cannot be regenerated is not a disadvantage. The exhausted adsorbent can be directly incinerated or used as foaming agent to obtain ceramic or glass foams.

## 4. Conclusions

The lignocellulosic material, bilberry (*Vaccinium myrtillus* L.) leaves powder, was used with high efficiency to remove the methylene blue dye from aqueous solutions. The pH, bioadsorbent dose, ionic strength, contact time, initial dye concentration, temperature influence the adsorption capacity and the dye removal efficiency. The adsorption is best described by Sips isotherm and general order kinetic model. The process is spontaneous, favorable and endothermic, involving a physisorption mechanism that can be enhanced by a small chemical effect. The factors with the higher percentages contribution on the dye removal efficiency were pH (53.34%) follow by contact time (22.11%) and temperature (12.00%). The material has a better absorption capacity compared to other similar adsorbents and has the advantage that it is easily available in nature, in large quantities, at a low cost.

## Figures and Tables

**Table 1:** Controllable factors and their levels used in L25 orthogonal array.

Factor	Level 1	Level 2	Level 3	Level 4	Level 5
pH	2	4	6	8	10
Time (min)	5	15	30	40	50
Adsorbent dose (mg·L^−1^)	1	2	3	4	5
Initial dye concentration (mg·L^−1^)	25	50	100	150	200
Temperature (K)	278	287	297	305	311
Ionic strength (mol L^−1^)	0	0.05	0.10	0.15	0.20

**Table 2:** The equilibrium times obtained for methylene blue adsorption on different similar bioadsorbent materials.

Adsorbent	Equilibrium Time (min)	Reference
*Humulus japonicas* leaves	20	[24]
*Daucus carota* leaves powder	30	[38]
*Arthrospira platensis* biomass	30	[25]
***Vaccinium myrtillus* L. leaves powder**	**40**	**This study**
*Archidendron jiringa* seed shells	60	[70]
*Typha angustifolia* leaves	60	[23]
pineapple leaf powder	60	[29]
*Platanus orientalis* leaf powder	70	[28]
*Phragmites australis* biomass	90	[71]
nonliving lichen *Pseudevernia furfuracea*	90	[72]
*Carica papaya* wood	100	[73]
*Ginkgo biloba* leaves	100	[74]
phoenix tree’s leaves	150	[30]
lotus leaf	150	[62]
*Citrullus colocynthis* peels	180	[1]

**Table 3:** The kinetic models constants and the corresponding error functions.

Kinetic Model	Parameters	Value
Pseudo-first order	*k*_1_ (min^−1^)	0.278 ± 0.032
	*q_e,calc_* (mg g^−1^)	20.25 ± 0.31
	*R* ^2^	0.9952
	*χ* ^2^	0.0893
	SSE	1.69
	ARE (%)	13.07
Pseudo-second order	*k*_2_ (min^−1^)	0.019 ± 0.008
	*q_e,calc_* (g mg^−1^ min^−1^)	21.48 ± 0.19
	*R* ^2^	0.9997
	*χ* ^2^	0.0050
	SSE	0.09
	ARE (%)	0.51
Elovich	*a* (g mg^−1^)	0.252 ± 0.047
	*b* (mg g^−1^ min^−1^)	2346 ± 127
	*R* ^2^	0.9936
	*χ* ^2^	0.1473
	SSE	2.26
	ARE (%)	13.30
General order	*k_N_* (min^−1^ (g mg^−1^)^n–1^)	4.256 ± 0.245
	*q_n_* (mg g^−1^)	21.27± 0.35
	*n*	1.269
	*R* ^2^	0.9999
	*χ* ^2^	0.0007
	SSE	0.01
	ARE (%)	0.16
Avrami	*k_AV_* (min^−1^)	0.636 ± 0.054
	*q_AV_* (mg g^−1^)	20.25 ± 0.48
	*n_AV_*	0.436
	*R* ^2^	0.9952
	*χ* ^2^	0.0888
	SSE	1.69
	ARE (%)	11.10

**Table 4:** The adsorption isotherms models constants and the corresponding error functions.

Isotherm Model	Parameters	Value
Langmuir non-linear	*K_L_* (L mg^−1^)	0.015 ± 0.001
	*q_max_* (mg g^−1^)	180.1 ± 5.17
	*R* ^2^	0.9994
	*χ* ^2^	0.0993
	SSE	2.47
	ARE (%)	2.80
Freundlich non-linear	*K_f_* (mg g^−1^)	4.41 ± 0.81
	*1/n*	0.74 ± 0.04
	*R* ^2^	0.9977
	*χ* ^2^	0.3887
	SSE	10.13
	ARE (%)	5.18
Temkin non-linear	*K_T_* (L mg^−1^)	0.314 ± 0.057
	*b* (kJ g^−1^)	94.94 ± 4.69
	*R* ^2^	0.9695
	*χ* ^2^	9.6559
	SSE	131.51
	ARE (%)	33.60
Sips non-linear	*Q_sat_* (mg g^−1^)	200.4 ± 5.74
	*K_S_* (L mg^−1^)	0.014 ± 0.002
	*n*	0.9625
	*R* ^2^	0.9994
	*χ* ^2^	0.0784
	SSE	2.25
	ARE (%)	2.27
Redlich-Peterson non-linear	*K_RP_* (L g^−1^)	2.90 ± 0.65
	*a_RP_* (L mg^−1^)	0.028 ± 0.003
	β_RP_	0.87 ± 0.09
	*R* ^2^	0.9994
	*χ* ^2^	0.0827
	SSE	2.28
	ARE (%)	2.37

**Table 5:** The maximum adsorption capacities for a several similar biomaterials used for the methylene blue adsorption.

Adsorbent	Maximum Adsorption Capacity (mg g^−1^)	Reference
*Arthrospira platensis* biomass	312.5	[25]
guava leaf powder	295.04	[83]
lotus leaf	221.7	[62]
***Vaccinium myrtillus* L. leaves powder**	**200.4**	**This study**
*Syringa vulgaris* leaves powder	188.2	[84]
*Humulus japonicas* leaves	145.56	[24]
*Platanus orientalis* leaf powder	114.9	[28]
tea waste	113.14	[63]
*Cocos nucifera* leaf	112.35	[85]
banana leaves	109.9	[86]
*Typha angustifolia* leaves	106.75	[23]
*Elaeis guineensis* leaves	103.0	[22]
phoenix tree’s leaves	80.9	[30]
*Daucus carota* leaves powder	66.5	[38]
*Salix babylonica* leaves	60.9	[27]
*Phragmites australis* biomass	58.82	[71]
potato leaves powder	52.60	[87]
*Carica papaya* wood	32.25	[73]
*Ginkgo biloba* leaves	48.07	[74]
*Archidendron jiringa* seed shells	44.64	[70]
Neem leaf powder	19.6	[88]
*Citrullus colocynthis* seeds	18.83	[1]
*Citrullus colocynthis* peels	4.36	[1]

**Table 6:** The thermodynamic parameters for the methylene blue adsorption on bioadsorbent obtained from bilberry (*Vaccinium myrtillus* L.) leaves.

ΔG^0^ (kJ mol^−1^)					ΔH^0^ (kJ mol^−1^)	ΔS^0^ (J mol^−1^ K^−1^)
278 K	287 K	297 K	303 K	311 K		
−18.91	−20.19	−21.56	−22.23	−23.1	2.02	15.49

**Table 7:** The L25 orthogonal array used in the experiments and results obtained after each run.

pH	Adsorbent Dose	Ionic Strenght	Time	Initial Dye Concentration	Temperature	Dye Removal Efficiency	S/N Ratio
2	1	0	5	25	278	32.95	30.35
2	2	0.05	15	50	287	47.23	33.48
2	3	0.1	30	100	297	51.78	34.28
2	4	1.15	40	150	305	51.18	34.18
2	5	2	50	200	311	50.01	33.98
3	2	2	5	100	305	50.39	34.04
3	3	0	15	150	311	69.6	36.85
3	4	0.05	30	200	278	57.01	35.11
3	5	0.1	40	25	287	69.41	36.82
3	1	1.15	50	50	297	60	35.56
6	3	1.15	5	200	287	51.16	34.17
6	4	2	15	25	297	74.46	37.43
6	5	0	30	50	305	87.92	38.88
6	1	0.05	40	100	311	75.21	37.52
6	2	0.1	50	150	278	62.44	35.90
8	4	0.1	5	50	311	65.24	36.29
8	5	1.15	15	100	278	62.26	35.88
8	1	2	30	150	287	59.91	35.55
8	2	0	40	200	297	77.37	37.77
8	3	0.05	50	25	305	90.44	39.12
10	5	0.05	5	150	297	62.43	35.90
10	1	0.1	15	200	305	63.95	36.11
10	2	1.15	30	25	311	79.82	38.04
10	3	2	40	50	278	65.9	36.37
10	4	0	50	100	287	82.17	38.29

**Table 8:** Response table for signal-to-noise S/N ratios (larger is better).

Level	pH	Adsorbent Dose	Ionic Strenght	Time	Initial Dye Concentration	Temperature
1	33.26	35.02	36.43 *	34.16	36.36 *	34.73
2	35.68	35.85	36.23	35.96	36.12	35.67
3	36.79	36.16	35.89	36.38	36.01	36.19
4	36.92	36.26	35.57	36.54	35.68	36.47
5	36.95 *	36.30 *	35.48	36.57 *	35.43	36.54 *
Delta	3.69	1.27	0.95	2.42	0.93	1.81
**Rank**	**1**	**4**	**5**	**2**	**6**	**3**
Contribution (%)	53.34	6.05	3.63	22.11	2.86	12.00

## Data Availability

All the experimental data obtained are presented, in the form of table and/or figure, in the article.
